# Serum from Parkinson’s disease patients presents excess of protein halogenation and nitrosylation, with anomalous nitrosylation of serum α-synuclein as potentially etiological factor

**DOI:** 10.1186/1750-1326-8-S1-P18

**Published:** 2013-09-13

**Authors:** Emilio Fernandez, Jose-Manuel Garcia-Moreno, Angel Martin de Pablos, Jose Chacon

**Affiliations:** 1 Department of Medical Physiology, University of Seville, Seville, Spain; 2 Hospital Macarena SAS, Seville, Spain; 3 Department of Surgery, University of Seville, Seville, Spain

## Background

Halogenative and nitrosative stress are two types of oxidative stress that have been proposed as pathogenic mechanisms in Parkinson’s disease (PD). They can be caused by over-stimulation of phagocytes. We hypothesize that maintained phagocyte overstimulation leads to both halogenative and nitrosative stress in Parkinson’s disease, which are present in serum and cerebrospinal fluid of patients. These types of oxidative stress could modify proteins related to the pathogenesis of Parkinson’s disease.

## Materials and methods

Halogenative and nitrosative stress-induced protein changes in serum and CSF were analyzed in PD patients (n=54) and control subjects without any neurological disorder (n=40), by using ELISA, western-blotting, mass spectrometry, culture techniques and immunofluorescence.

## Results

In our lab, it has been detected the presence of halogenative stress in serum and, to a lesser extent, cerebrospinal fluid of Parkinsonian patients leading to excess of advanced oxidized protein products or AOPP. On the other hand, nitrosative stress is also present in serum and cerebrospinal fluid of patients with early PD, characterized by the selective increase of 3-nitrotyrosine proteins other than nitroalbumin and free 3-nitrotyrosine. Nitrosylation stress is accompanied by modification of the sites of nitrosylation of α-synuclein in patients with early PD, characterized by dominant nitrosylation of tyrosine 125/136 residues. *In vitro* studies revealed that 60K-filtrated serum from PD patients is cytotoxic for substanta nigra neurons in culture, and it is able to induce proteinaceous aggregates inside these neurons, which are strongly positive to nitro-α-synuclein. Interestingly, cytotoxicity can be blocked by using antibodies aimed to nitrosylated tyrosine-39 of the molecule.

## Conclusions

Since metabolism of advanced oxidized protein products and 3-nitrotyrosine proteins has been associated to phagocytic overstimulation, this pathological alteration could play a pathogenic role in sporadic PD. Our observations also lead to the hypothesis that serum level of advanced oxidized protein products is a prognostic marker for Parkinson’s disease duration, and these oxidized proteins could participate in neuroinflammation. Besides, the evaluation of nitrosative stress through enhanced levels of 3-nitrotyrosine proteins in serum and cerebrospinal fluid without changes in nitroalbumin, together with the profile of tyrosine nitrosylation of serum α-synuclein characterized by dominant nitrosylation of Tyr125/136 could serve for diagnosis of sporadic Parkinson’s disease. Nitro-α-synuclein is a main component of Lewy bodies, hallmarks of the disease, and serum nitro-α-synuclein could represent a pathogenic factor in PD. Finally, we have *in vitro* evidence that blockade of tyrosine residue 39 of nitro-α-synuclein could abolish cytotoxicity of Parkinsonian serum.

